# Évaluation du coût du traitement des envenimations par morsure de serpent à l'hôpital Saint Jean de Dieu de Tanguiéta, Bénin

**DOI:** 10.48327/mtsi.v4i4.2024.522

**Published:** 2024-12-10

**Authors:** Naryanan TOURITA, Noé SODJINOU, Seidou Alassane OUOROU, Éric GANHOUINGNON, Achille MASSOUGBODJI, Jean-Philippe CHIPPAUX, Sébastien LARRÉCHÉ

**Affiliations:** 1 Département Santé, Université Senghor, 1, Place Ahmed Orabi, Al Mancheya, BP 415, 21111 Alexandrie, Égypte; 2 Institut de recherche clinique du Bénin, Abomey-Calavi, Bénin; 3 Service des urgences, Hôpital Saint-Jean de Dieu, Tanguiéta, Bénin; 4 Laboratoire de biologie, Hôpital Saint-Jean de Dieu, Tanguiéta, Bénin; 5 Institut de recherche clinique du Bénin, Abomey-Calavi, Bénin; 6 Institut de recherche pour le développement, Unité de recherche MERIT, Université Paris Cité, 4 avenue de l’Observatoire, F-75006 Paris, France; 7 Service de biologie médicale, Hôpital national d'instruction des armées Bégin, 69 avenue de Paris, 94160 Saint-Mandé; UMR-S1144, Université Paris Cité, 4 avenue de l’Observatoire, 75006, Paris, France

**Keywords:** Antivenin, Coût du traitement, Envenimations, Morsures de serpent, *Echis ocellatus*, Bénin, Afrique subsaharienne, Antivenom, Treatment costs, Envenomations, Snakebites, *Echis ocellatus*, Benin, Sub-Saharan Africa

## Abstract

**Introduction:**

Les envenimations par morsure de serpent sont un réel problème de santé publique dans les zones rurales d’Afrique subsaharienne, d'autant que le coût des soins de cette pathologie est souvent hors de portée des victimes. L'objectif de notre étude était d’évaluer le coût de la prise en charge des envenimations par morsure de serpent à l'hôpital Saint Jean de Dieu de Tanguiéta, au Nord Bénin, en zone de savane.

**Méthodes:**

Il s'agissait d'une étude transversale descriptive, s’étalant sur une période de trois mois, allant du 25 mai au 25 août 2023. Le suivi des patients mordus par un serpent a été effectué depuis l'admission jusqu’à la sortie de l'hôpital. Toutes les dépenses liées aux soins assumées par les patients et/ou leurs familles ont été comptabilisées de façon journalière.

**Résultats:**

Cinquante-sept patients ont été inclus. L’âge médian (interquartiles) des patients était de 27 ans (16-40), et le sex-ratio de 1,6 (35 hommes et 22 femmes). Dans 81 % des cas, ces morsures étaient liées aux activités agricoles. Environ 72 % des patients avaient eu recours aux soins traditionnels avant de se rendre à l'hôpital. Le délai médian morsure - admission était de 7 heures (2-52) et la durée médiane de séjour à l'hôpital de 4 jours (2-5). Le coût médian de la prise en charge était de 168 € (154-242). Il variait selon la sévérité de la morsure : 31 € pour une morsure sèche (26-47); 179 € en cas de saignement extériorisé (154-286). Le seul antivenin utilisé était l'inoserp^TM^ PAN-AFRICA (Inosan Biopharma). Son coût moyen était de 128 € et constituait la principale dépense.

**Conclusion:**

Le coût du traitement des envenimations par morsure de serpent est élevé et dominé par celui de l'antivenin. Ces contraintes économiques renforcent le cercle vicieux de la pauvreté dans une population déjà précaire. Il est donc important de trouver un mécanisme de financement de ce traitement dans les zones les plus exposées.

## Abstract (en)

**Introduction:**

Snakebite envenomations are a real public health problem in rural areas of sub-Saharan Africa, especially as the cost of management is often beyond the reach of the victims. The aim of our study was to evaluate the costs of treating snakebite envenomations at the Saint Jean de Dieu hospital in Tanguiéta, northern Benin, in a savannah area.

**Methods:**

This was a descriptive cross-sectional study conducted over a three-month period from May 25 to August 25, 2023. Snakebite patients were followed from admission to hospital discharge. All care-related expenses incurred by patients and/or their families were accounted for on a daily basis.

**Results:**

Fifty-seven patients were included. The median age (interquartile range) of the patients was 27 years (16-40), and the sex ratio was 1.6 (35 males and 22 females). In 81% of cases, the bites were associated with agricultural activities. Approximately 72% of the patients had sought traditional care before coming to the hospital. The median time from bite to admission was 7 hours (2-52) and the median hospital stay was 4 days (2-5). The median cost of care was €168 (154-242). It varied according to the severity of the bite: €31 for a dry bite (26-47); €179 for external bleeding (154-286). The only antivenom used was Inoserp™ PAN-AFRICA (Inosan Biopharma). Its average cost was €128 and was the main expense.

**Conclusion:**

The cost of treating snakebite is high and dominated by the cost of antivenom. These economic constraints reinforce the vicious circle of poverty in an already vulnerable population. It is therefore important to find a financing mechanism for this treatment in the most exposed areas.

## Introduction

Chaque année en Afrique subsaharienne, plus de 300 000 victimes de morsures de serpent consultent dans un centre de santé, avec 7 300 décès et environ 10 000 invalidités définitives [2]. La plupart des patients sont de jeunes actifs, travailleurs ruraux [1]. La prise en charge est assumée financièrement par les patients et leur famille. Or l'antivenin, qui constitue le pilier de la prise en charge, a un coût élevé. Il en résulte un retentissement économique et social important. Bien qu'il s'agisse d'une urgence médicale, l'administration de l'antivenin peut être retardée ou non réalisée, faute de moyens financiers. Néanmoins, le coût global de la prise en charge de cette pathologie a été peu étudié. Ainsi, nous avons voulu évaluer le coût du traitement des envenimations par morsure de serpent à l'hôpital Saint Jean de Dieu de Tanguiéta, au Nord Bénin, afin d'estimer le fardeau économique de cette pathologie.

Notre objectif était de mesurer le coût global du traitement des envenimations. Plus spécifiquement, nous avons voulu déterminer les coûts de chaque composante du traitement (antivenin, traitement adjuvant, hospitalisation, bilan biologique), la durée de séjour à l'hôpital, la proportion des patients ayant eu recours à la médecine traditionnelle avant consultation à l'hôpital et l'impact de la gravité sur le coût du traitement.

## Matériel et méthodes

### Cadre d’étude

L'hôpital Saint Jean de Dieu de Tanguiéta est un hôpital confessionnel situé dans l’Atacora, une région montagneuse au nord-ouest du Bénin. Il sert d'hôpital de première référence et dessert une zone ayant une superficie de 7 900 km^2^ avec une population estimée à 320 102 habitants en 2023. L'hôpital enregistre chaque année plus de 100 cas de morsures de serpent, le plus souvent causées par la vipère *Echis ocellatus* [1].

Les frais liés aux soins sont assumés par les patients et leur famille, selon la tarification à l'acte en plus des frais d'hospitalisation. Le coût de l'acte varie selon le traitement (médicaments prescrits, examens biologiques).

À l'admission, une fois le diagnostic d'envenimation retenu, le médecin prescrit l'antivenin Inoserp™ PAN-AFRICA (Inosan Biopharma, Mexico, Mexique) conformément au protocole du ministère de la santé publique du Bénin. Cet antivenin est lyophilisé. Il est administré en injection intraveineuse directe lente. La dose initiale est d'une ampoule en cas d'atteinte locale isolée (marquée notamment par un œdème), deux ampoules si des saignements sont présents ou si le test de coagulation sur tube sec (TCTS) est anormal, enfin quatre ampoules s'il y a des signes neurologiques d'envenimation par Elapidae (ptosis, dyspnée…) [3]. Chaque ampoule coûtant 64 €, l'antivenin a donc un coût initial de 64 à 256 €. Ce traitement est renouvelé deux heures plus tard en cas de persistance du saignement ou d'un signe neurologique, à raison de 2 ou 4 ampoules respectivement, soit 128 à 256 € supplémentaires. Ce traitement est renouvelé toutes les deux heures, jusqu’à ce que les signes s'améliorent. À côté du traitement antivenimeux, les patients bénéficient des examens de biologie médicale (numération formule sanguine, goutte épaisse pour recherche de paludisme, groupage sanguin, voire d'autres examens en fonction de la présentation clinique du patient) et de traitements adjuvants (sérum antitétanique, antibiotiques, antalgiques.). Chaque composante de ce traitement a un coût, de même que l'hospitalisation. Tous les médicaments sont achetés à la pharmacie de l'hôpital, à partir d'un numéro d'identification personnel unique pour chaque patient. Ce numéro est attribué par le service de comptabilité de l'hôpital et sert à la facturation des actes et frais hospitaliers. Toutes les données financières sont centralisées au niveau du service d'administration et de la comptabilité. Une souche de la facture est toujours remise au patient.

### Méthode de collecte et d'analyse des données

Nous avons collecté les données sur une période de trois mois, du 25 mai au 25 août 2023 à l'hôpital Saint Jean de Dieu de Tanguiéta. Tout en assurant le suivi médical, nous avons notifié toutes les dépenses effectuées par les patients mordus par un serpent.

L'interrogatoire et l'examen physique ont permis de connaître les circonstances de morsure, les facteurs associés (distance entre lieu géographique de morsure et hôpital, recours aux soins traditionnels), de confirmer une envenimation et de préciser le niveau de gravité. Un test de coagulation sur tube sec (TCTS) a été systématiquement réalisé à l'entrée afin de rechercher une éventuelle altération de l'hémostase, classiquement associée à l'envenimation par *E. ocellatus* [4]. Chaque patient a été examiné toutes les deux heures jusqu’à H8, puis à H12, H24 et quotidiennement jusqu’à sa sortie, sauf situation particulière nécessitant un suivi plus fréquent. Lors de la surveillance du patient, nous avons vérifié, avec son consentement, la facture de toutes les dépenses effectuées depuis son admission. À sa sortie, un numéro d'identification personnel a été envoyé au service de comptabilité de l'hôpital qui récapitulait et mettait à jour le relevé de toutes les dépenses effectuées par le patient. Nous avons confronté ce relevé avec nos propres données obtenues auprès du patient pour nous assurer de leur concordance. Nous avons classé ces dépenses par rubrique : hospitalisation, antivenin, traitement adjuvant, examens de biologie médicale.

Différentes variables ont été notifiées : données cliniques et biologiques, délai d'admission après la morsure, recours à la médecine traditionnelle, nombre d'ampoules d'antivenin utilisées, coût du traitement, proportion des dépenses pour chaque élément de la prise en charge (antivenin, traitement adjuvant, examens de biologie médicale, hospitalisation), durée de séjour à l'hôpital. Nous avons ordonné les morsures de serpent selon le degré de gravité, à partir de l'examen clinique et du TCTS réalisés à l'admission. Cette classification détermine la conduite thérapeutique à tenir. Toute morsure de serpent asymptomatique était considérée comme une morsure sèche (c'est-à-dire sans injection de venin) ne requérant pas d'antivenin. Les cas d'envenimation avérée étaient classés en trois catégories : syndrome inflammatoire local isolé sans trouble de la coagulation, anomalie du TCTS sans hémorragie et syndrome hémorragique lorsqu'un saignement, quelle que soit sa localisation, se prolongeait au-delà de 30 minutes.

### Considérations éthiques

Les données ont été recueillies après consentement informé des patients. Les données financières fournies par le service de comptabilité ont été vérifiées par le médecin référent en charge des envenimations, avant toute exploitation. Il s'agit d'une étude ancillaire réalisée dans le cadre d'un essai clinique (étude ACTRASES) qui a reçu le 25 juillet 2022 un avis favorable du Comité national d’éthique pour la recherche en santé du ministère de la Santé de la République du Bénin (n°101/MS/DC/SGM/CNERS/SA).

### Analyse statistique

Les données ont été saisies sur une base Microsoft Excel 2019, puis analysées à l'aide du logiciel R version 4.1.2. Les variables qualitatives ont été exprimées sous forme de pourcentages et les variables quantitatives sous forme de médiane, assortie des quartiles à 25 % et 75 %. La normalité des variables quantitatives a été vérifiée par le test de Shapiro-Wilk. Du fait d'une distribution asymétrique de ces variables, les groupes de patients ont été comparés par un test de Kruskall-Wallis suivi par un test de comparaisons multiples de Dunn.

## Résultats

Nous avons inclus 57 patients dans cette étude.

### Données démographiques

L’âge médian (interquartiles) des patients mordus a été de 27 (16-40) ans. Les hommes ont constitué 61 % des patients et les femmes mordues 39 %; les tranches d’âge les plus représentées ont été celles de 11-20 ans et de 21-30 ans (Tableau I).

**Tableau I:** Répartition des patients par tranche d’âge et par genre

Données démographiques	Effectifs (N=57)	%
Tranche d’âge (an)		
1 - 10	7	12
11 - 20	11	19
21 - 30	17	30
31 - 40	9	16
41 - 50	9	16
51 - 60	4	7
Genre		
homme	35	61
femme	22	39
**Total**	**57**	**100**

### Circonstances de morsure et recours aux soins traditionnels

Sur les 57 patients, 41 (72 %) ont eu recours aux soins traditionnels avant de se rendre à l'hôpital, les 16 autres (28 %) se sont rendus directement à l'hôpital.

**Figure 1:**
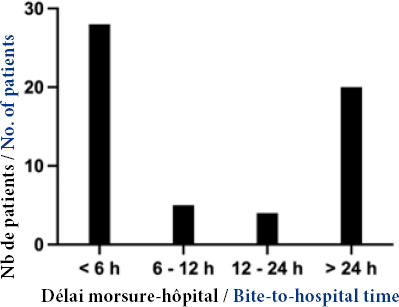
Distribution des patients en fonction du délai entre morsure et admission à l'hôpital

La répartition des patients en fonction du délai de prise en charge est présentée Figure 1. Le délai médian morsure – admission a été de 7 heures (IQ : 2 – 48). La majorité des morsures (35/57, soit 65 %) sont survenues dans les champs.

Le siège des morsures était réparti comme suit : 47 (82 %) au pied, 8 (14 %) à la main, 1 (2 %) au bras et 1 (2 %) à la fesse.

### Classification selon la sévérité

Nous n'avons pas observé de patient présentant des troubles neurologiques pouvant faire suspecter une envenimation par un élapidé.

Les différentes atteintes cliniques sont présentées dans le Tableau II. Les cas de syndrome hémorragique ont été les plus fréquents et traduisaient une envenimation vipérine.

**Tableau II:** Classification et durée médiane d'hospitalisation des envenimations selon le degré de sévérité

Classification	Fréquence (N=57)	Durée médiane (IQ) d'hospitalisation (jours)
Morsure sèche	**7 (12)**	**1 (1 -1)**
Syndrome inflammatoire local isolé	**15 (26)**	**3 (2 – 4)**
Anomalie du TCTS sans saignement	**11 (20)**	**4 (3 – 8)**
Saignement	**24 (42)**	**4 (3 – 6)**

La durée médiane d'hospitalisation en fonction de la forme clinique est présentée dans le Tableau II.

La durée maximale d'hospitalisation a été de 11 jours. La forme clinique a influencé la durée d'hospitalisation (p = 0,003) qui a été plus importante en cas d'anomalie du TCTS sans saignement, ou de saignement en comparaison avec une morsure sèche (respectivement p = 0,01 et p = 0,007). En revanche, il n'y a pas eu de différence entre les durées d'hospitalisation pour une anomalie du TCTS sans saignement et pour un saignement (p > 0,99).

Sur un total de 57 patients, nous avons observé deux décès (3 %), liés à une hémorragie : le premier cas a été une patiente de 25 ans qui s'est présentée aux urgences une semaine après la morsure de serpent; le décès est survenu 30 minutes après son admission à l'hôpital. Le 2^e^ cas, survenu 2 heures après l'admission à l'hôpital, a concerné une patiente de 17 ans, enceinte de 20 semaines, admise 2 heures après une morsure par *Bitis arietans* (un Viperidae plus grand qu’*E. ocellatus)* malgré l'administration d'antivenin.

### Coût du traitement

Au total, 108 ampoules d'antivenin ont été utilisées pour traiter 45 patients (Tableau III). Douze patients (sept morsures sèches et cinq syndromes inflammatoires avec un œdème local) ont seulement bénéficié d'un traitement symptomatique, car leur état clinique ne nécessitait pas d'antivenin. Les cas de syndrome hémorragique ou d'anomalie du TCTS ont nécessité parfois quatre à six ampoules d'antivenin.

**Tableau III:** Nombre d'ampoules d'antivenin utilisées et coût médian selon la sévérité d'envenimation

Classification	Nb de patients mordus	Nb d'ampoules utilisées	Nb médian d'ampoules (IQ)	Coût médian (IQ) en euros
Morsure sèche	7	0	0 (0 - 0)	31 (26 - 47)
Syndrome inflammatoire local isolé	15	25	2 (2 - 2)	163 (149 - 170)
Anomalie du TCTS sans saignement	II	24	2 (2 - 2)	177 (161 - 327)
Saignement	24	59	2 (2 - 2)	179 (154 - 286)
**Total**	**57**	**108**	**2 (2 - 2)**	

Le coût médian en fonction de la sévérité est présenté dans le Tableau III. Nous avons observé une différence significative pour le coût du traitement en fonction de la forme clinique (p = 0,0001) : comparé à une morsure sèche, le coût des formes symptomatiques s'est révélé plus élevé pour un syndrome inflammatoire local isolé (p > 0.04), une anomalie du TCTS sans saignement (p = 0,0005) ou un saignement (p = 0,0002). Il n'a pas été noté de différence significative entre les coûts médians des trois formes symptomatiques d'envenimation (syndrome inflammatoire local isolé, anomalie du TCTS sans saignement et saignement). L'antivenin a constitué la principale dépense pour la prise en charge des patients (Tableau IV). Pour chacun d'eux, l'antivenin a représenté un coût plus élevé que les traitements complémentaires (p = 0,0001), l'hospitalisation (p > 0,0001) et les examens de biologie médicale (p > 0,0001). À l’échelle d'un patient, l'antivenin correspondait à environ 75 % du coût total du traitement.

**Tableau IV:** Coût du traitement, total et médian par patient

Actes	Coût total (IQ) en euros	Coût médian (IQ) par patient en euros
Antivenin	7 115	128 (128 – 128)
Traitement complémentaire	1 264	15 (10 – 30)
Hospitalisation	796	12 (8 – 20)
Examens de biologie médicale	817	11 (11 – 15)
**Total**	**9 992**	**168 (154 – 242)**

## Discussion

Cette étude prospective présente une série de patients dont la prise en charge a strictement respecté le protocole thérapeutique, procurant ainsi une base solide d'estimation du coût du traitement des envenimations par morsure de serpent à l'hôpital de zone Saint Jean de Dieu de Tanguiéta.

Les coûts directs représentent ceux des soins, des consultations, des médicaments, de l'hospitalisation, des frais de transport, etc. Les coûts indirects peuvent être la perte de productivité due au temps d'hospitalisation, pour les malades et leurs familles ainsi que pour leur pays du fait du nombre important d'envenimations. Il y a peu d’études effectuées en milieu rural africain qui se sont intéressées à la charge économique des envenimations [9, 10] et aucune, à notre connaissance, au coût direct de leur prise en charge à l’échelle du patient. Notre étude s'est déroulée dans une zone endémique de morsure par *E. ocellatus,* durant la période de l'année qui enregistre le plus de cas [1]. Elle a été menée uniquement au niveau de l'hôpital et n'a concerné que les tout premiers jours de l'envenimation dont les effets peuvent se prolonger plusieurs semaines.

Le coût élevé, variable en fonction de l'antivenin disponible, n'est que rarement à la portée des populations rurales d’Afrique subsaharienne [6, 11, 13, 14, 15, 16]. Dans notre étude, l'immunothérapie antivenimeuse a coûté en moyenne 128 € constituant 76 % du traitement. Au Bénin, où le salaire mensuel minimum est de 78 € [18] et le revenu mensuel moyen par habitant de 95 € en 2018 [20], le coût du traitement hospitalier correspondait presqu’à deux fois le revenu mensuel moyen par habitant. Néanmoins, tous les patients dont l’état nécessitait l'antivenin l'ont reçu. L'hôpital de Tanguiéta est un hôpital confessionnel et accorde des facilités de paiement des frais engagés pour la prise en charge de l'envenimation par le patient et sa famille. Ce dispositif permet d'assurer un traitement conforme au protocole thérapeutique en limitant les conséquences financières.

En Afrique subsaharienne, c'est le patient et sa famille qui doivent assurer la totalité des dépenses avant de bénéficier des soins, ce qui prolonge souvent le délai de prise en charge. Le coût du traitement des envenimations est souvent difficile à assumer par le patient et sa famille [12]. Dans une étude au Nigeria, une famille a déclaré que le remboursement de la dette contractée lui prendrait une année entière tandis que d'autres familles se sont vues dans l'obligation de vendre du bétail pour financer les soins [8]. Cet impact économique aggrave la pauvreté qui, elle-même, est un facteur de mortalité accrue, conduisant à un véritable cercle vicieux [19]. C'est durant ou après l'hospitalisation, quand les patients sont sur le point de rentrer chez eux, qu'ils font de leur mieux pour s'acquitter de la totalité de ces frais. Outre les raisons culturelles et mystiques guidant le parcours thérapeutique d'une victime de morsure de serpent, les conséquences financières et sociales influent fortement sur le retard, voire le refus, de consulter dans un centre de santé. Okumu *et al.,* dans une étude rétrospective menée dans un hôpital de référence au Kenya, notent que le coût médian du traitement était de 26 dollars (24 €) [14]. Mais ce coût plutôt bas était lié à une fréquente rupture d'antivenin au niveau de cet hôpital. Sur 127 patients mordus, seuls 42 % d'entre eux avaient reçu un antivenin, généralement à la dose d'une ampoule. À Tanguiéta, durant la période d’étude, aucune rupture de l'approvisionnement en antivenin n'a été signalée. L’étude d’Okumu *et al.* note aussi que le prolongement du temps d'hospitalisation était associé à une augmentation du coût de traitement [14]. Ceci peut aussi être une incitation à quitter l'hôpital contre avis médical, alors que l’état du patient nécessite encore des soins. Le temps d'hospitalisation, et donc le coût, peuvent être majorés en cas d'envenimation sévère. Cependant, dans notre étude, il n'y a pas eu de différence significative entre les coûts correspondant aux différentes formes cliniques. En contrepartie, il doit être noté que l'algorithme préconisé par la Société africaine de venimologie ne prévoit qu'une seule ampoule d'antivenin en cas de syndrome local isolé alors que les patients ont reçu deux ampoules, ce qui a majoré inutilement le coût de la prise en charge. Le fardeau total des envenimations en Afrique de l’Ouest a été estimé à 320 000 DALYs *(disability-adjusted life years*; espérance de vie corrigée de l'incapacité) /an (IC 95% : 248 000-403 000) [9]. Avec un rapport coût-efficacité élevé de l'utilisation des antivenins, il est primordial pour les décideurs et les gouvernements des pays d’Afrique subsaharienne de mettre en place un mécanisme de réduction des prix ou de compensation financière [7, 11, 15, 17]. Selon O’Brian *et al.,* au Mozambique, le coût individuel médian des morsures de serpent dû aux pertes de revenus qui s'en suit est de 17 dollars – non compris le traitement, notamment l'antivenin qui n'a pas été chiffré dans cette étude –, cinq fois plus élevé que le coût du traitement d'un cas de paludisme simple [13]. Cependant, les envenimations suscitent peu d'attention de la part des décideurs. Au niveau du Bénin ainsi que dans d'autres pays d’Afrique subsaharienne, il existe des mécanismes de gratuité des soins pour certaines éventualités (paludisme, césarienne, etc.) que l'on pourrait étendre aux envenimations.

En attendant la gratuité des soins prônée par plusieurs auteurs [7, 16], on peut proposer une répartition équitable des coûts en s'appuyant sur deux leviers. D'une part, l'approvisionnement en antivenins dont le coût pourrait être réparti entre l’État qui, en plus d'une subvention, centraliserait les commandes pour bénéficier d'une économie d’échelle, les collectivités locales qui assureraient l'estimation des besoins et la gestion des stocks régionaux, les entreprises privées qui contribueraient au règlement de la facture, pour laisser au patient un reste à charge supportable. D'autre part, la facturation de l'hôpital pourrait être forfaitaire, partageant les charges entre les différents patients indépendamment de la gravité de leur envenimation.

## Limitations

Dans notre étude, nous nous sommes limités aux coûts directs médicaux. Il nous aurait été difficile d’évaluer les coûts directs non médicaux (frais des transports, restauration, etc.). Par exemple, les patients ont été acheminés à l'hôpital avec des moyens de transport personnels, le plus souvent une moto, ou empruntés – peut-être loués – dans la communauté. Notre étude a mesuré uniquement les dépenses effectuées par les malades. Il serait intéressant de montrer les dépenses réelles supportées par l'hôpital (coût du sérum antivenimeux, autres médicaments, frais de restauration, paiement du personnel…).

En outre, les coûts indirects (par exemple absence au travail) supportés par les accompagnateurs des patients n'ont pu être évalués du fait de la difficulté de la tâche; ils sont nombreux à laisser leur travail pour être aux côtés des malades (solidarité communautaire). L'absentéisme d'un membre de la communauté entraîne une réorganisation, au sein de la famille ou du village, du partage des activités [5]. L'entraide permet une suppléance non onéreuse dont l'objectif est d’éviter le recours à une main-d’œuvre de substitution salariée. Encore très active entre les membres de la communauté, elle contribue à réduire les charges représentées par les coûts indirects.

Cette étude a été menée en marge d'un essai clinique de phase 4 ayant pour objectif d’évaluer l'efficacité et la tolérance de l'acide tranexamique en association à l'antivenin dans le traitement des saignements associés aux envenimations par morsure de serpent (étude ACTRASES). Parmi les 45 patients traités avec l'antivenin, 14 ont été inclus dans cet essai et ont ainsi pu bénéficier d'une prise en charge de la plus grande partie de leurs frais. Il est donc possible que cela ait artificiellement majoré dans notre étude le poste représenté par les antivenins qui n'auraient peut-être pas été achetés si le patient avait dû les payer. Enfin, cette étude n'a pas permis de décrire les traitements traditionnels reçus par les patients ni d'en évaluer les éventuels bénéfices et risques. Outre le retard de prise en charge dû à la consultation d'un thérapeute traditionnel, les gestes pratiqués par ce dernier comme la pose d'un garrot, les incisions cutanées ou l'administration de plantes émétiques pour ralentir la diffusion du venin ou favoriser son élimination, pourraient être délétères et entraîner un prolongement du temps d'hospitalisation et un surcoût de la prise en charge.

## Conclusion

Notre étude nous a permis de confirmer que les envenimations par morsure de serpent ont un coût élevé, parfois supérieur au revenu mensuel moyen de nombreux ménages. En cas d'hémorragie, le coût est encore augmenté du fait du renouvellement des ampoules d'antivenin. Ces morsures surviennent en milieu rural, dans une population pauvre, qui pratique les activités agropastorales, avec des moyens rudimentaires. Elles affectent les jeunes qui représentent la couche la plus productive de la société. Liées aux activités agricoles, les envenimations ophidiennes les compromettent, entraînant une baisse de productivité et un risque supplémentaire d'appauvrissement. Le traitement est entièrement supporté par les patients et leurs familles, ce qui renforce le cercle vicieux de la pauvreté.

Il est donc souhaitable de mettre en place un mécanisme de financement du traitement des envenimations. À défaut de mettre les antivenins gratuitement à la disposition des patients, une répartition de leur coût entre toutes les parties prenantes pourrait être envisagée. La diminution du coût de l'antivenin permettra un plus grand accès des patients à l'hôpital et une prise en charge précoce des patients, conduisant ainsi à diminuer le fardeau économique des envenimations.

## Remerciements

Nous remercions l'ensemble du personnel de l'hôpital Saint Jean de Dieu de Tanguiéta pour son accueil et son aide au cours de cette étude. Notre gratitude va également aux patients et à leur famille pour leur participation et leur bienveillante compréhension lors de nos interrogatoires. Merci aussi à l’Institut de recherche clinique du Bénin qui nous a permis de mener cette étude simultanément à l'essai clinique ACTRASES.

## Financement

Le financement de cette étude a été partagé entre l’Institut de Recherche pour le Développement et l’Institut de Recherche Clinique du Bénin (logistique), l’Université Senghor d’Alexandrie, Egypte) (prise en charge de N. Tourita) et Inosan Biopharma (fourniture de l'antivenin dans le cadre de l’étude ACTRASES).

## Contribution des auteurs

NT : conception de l’étude, rédaction du protocole, recueil des données, analyse des données, rédaction du manuscrit

NS : recueil des données, correction du manuscrit

SAO : recueil des données, vérification et validation des résultats, correction du manuscrit

EG : recueil des données, correction du manuscrit

AM : promoteur de l’étude, révision et validation du protocole, correction du manuscrit

JPC : acquisition du financement, révision et validation du protocole, validation des données, validation de l'analyse, révision et correction du manuscrit

SL : conception de l’étude, rédaction et validation du protocole, coordination de l’étude, vérification et validation des données, vérifications de l'analyse, révision et correction du manuscrit

## Liens d'intérêts

Les auteurs déclarent ne pas avoir de liens d'intérêts.
