# Supplementary material for: Evaluation of snakebite management cost at Saint Jean de Dieu Hospital in Tanguiéta, Benin
Source: Med Trop Sante Int. 2024 Dec 10;4(4):mtsi.v4i4.2024.522. [Article in French] doi: 10.48327/mtsi.v4i4.2024.522 (PMC11895845; doi:10.48327/mtsi.v4i4.2024.522)
Supplement: Related texts

# Research article: Evaluation of snakebite management cost at Saint Jean de Dieu Hospital in Tanguiéta, Benin

## Introduction

Each year in sub-Saharan Africa, more than 300.000 snakebite victims present to a health facility, resulting in 7,300 deaths and approximately 10.000 permanent disabilities [2]. Most patients are young, active rural workers [1]. The cost of treatment is covered by patients and their families. However, antivenom, the mainstay of treatment, is expensive. This has a significant economic and social impact. Although this is a medical emergency, the administration of antivenom may be delayed or withheld due to lack of financial resources. However, the total cost of managing this pathology has been little studied. We therefore set out to evaluate the costs of treating snakebite envenomations at the Saint Jean de Dieu Hospital in Tanguiéta, northern Benin, in order to estimate the economic burden of this pathology.

Our objective was to measure the total cost of treating envenomations. More specifically, we wanted to determine the cost of each component of treatment (antivenom, adjuvant treatment, hospitalization, biological work-up), the length of hospital stay, the proportion of patients who resorted to traditional medicine before hospital consultation, and the impact of severity on the cost of treatment.

## Materials and methods

### Study Setting

The Saint Jean de Dieu Hospital in Tanguiéta is a faith-based hospital located in Atacora, a mountainous region in northwestern Benin. It serves as the primary referral hospital for an area of 7,900 km^2^ with an estimated population of 320,102 in 2023. Each year, the hospital registers more than 100 cases of snakebite, most commonly caused by the *Echis ocellatus* viper [1].

The cost of treatment is covered by patients and their families on a fee-for-service basis, in addition to hospital fees. The cost of the procedure varies according to the treatment (prescribed drugs, biological tests).

On admission, once the diagnosis of envenomation has been accepted, the doctor prescribes the antivenom Inoserp™ PAN-AFRICA (Inosan Biopharma, Mexico City, Mexico) in accordance with the protocol of the Ministry of Health of Benin. This antivenom is lyophilized. It is administered by slow, direct intravenous route.

The initial dose is one vial in case of isolated local involvement (characterized in particular by edema), two vials in case of bleeding or abnormal whole blood clotting test (WBCT), and four vials in case of neurological signs of Elapidae envenomation (ptosis, dyspnea…) [3]. Each vial costs €64, so the initial cost of antivenom is between €64 and €256. This treatment is repeated two hours later if bleeding or neurological signs persist, at a cost of 2 or 4 vials respectively, i.e. another €128 to €256. This treatment is repeated every two hours until signs improve.

In addition to the antivenom, patients benefit from medical-biological tests (blood count, thick blood smear for malaria, blood grouping and other tests depending on the patient's clinical presentation) and adjuvant treatments (anti-tetanus serum, antibiotics, analgesics, etc.). Each component of this treatment has a cost, as does hospitalization. All medications are purchased by the hospital pharmacy using a unique personal identification number for each patient. This number is assigned by the hospital's accounting department and is used for billing hospital procedures and charges. All financial information is centralized in the administration and accounting department. A copy of the bill is always given to the patient.

### Data Collection and Analysis Methods

We collected data over a three-month period, from May 25 to August 25, 2023, at the Hôpital Saint Jean de Dieu in Tanguiéta. While ensuring medical follow-up, we reported all expenses incurred by snakebite patients.

Through interviews and physical examinations, we were able to determine the circumstances of the bite, the associated factors (distance between the geographical location of the bite and the hospital, use of traditional medicine), confirm the envenomation and determine the severity. A whole blood clotting test (WBCT) was systematically performed on admission to look for any alteration in hemostasis, which is classically associated with *E. ocellatus* envenomation [4]. Each patient was monitored every two hours until H8, then at H12, H24, and daily until discharge, except in special situations requiring more frequent monitoring. During monitoring, we reviewed the patient's bill for all expenses incurred since admission, with the patient's consent. At discharge, a personal identification number was sent to the hospital's accounting department, which summarized and updated the statement of all expenses incurred by the patient. We compared this statement with our own data obtained from the patient to ensure consistency. We classified these expenses according to the following headings: hospitalization, antivenom, adjuvant treatment, medical-biological tests. Different variables were reported: clinical and biological data, admission time after the bite, use of traditional medicine, number of vials of antivenom used, cost of treatment, proportion of expenditure for each element of management (antivenom, adjuvant treatment, medical biology tests, hospitalization), length of hospital stay. We classified snakebites according to degree of severity, based on the clinical examination and WBCT performed on admission. This classification determines the therapeutic course of action to be taken. Any asymptomatic snakebite was considered a dry bite (i.e. without venom injection) and did not require antivenom. Cases of proven envenomation were classified into three categories: isolated local inflammatory syndrome without coagulation disorder, WBCT abnormality without hemorrhage, and hemorrhagic syndrome when bleeding, regardless of location, continued for more than 30 minutes.

### Ethical considerations

Data were collected with the informed consent of the patients. Financial data provided by the accounting department were verified by the referring physician in charge of envenomations, prior to any use. This is an ancillary study carried out as part of a clinical trial (ACTRASES study) which received a favorable opinion from the National Health Research Ethics Committee of the Ministry of Health of the Republic of Benin on July 25, 2022 (n°101/MS/DC/SGM/CNERS/SA).

### Statistical Analysis

Data were entered into a Microsoft Excel 2019 database and analyzed using R software, version 4.1.2. Categorical variables were expressed as percentages, and quantitative variables were expressed as medians, with quartiles at 25% and 75%. Normality of quantitative variables was tested using the Shapiro-Wilk test. Due to the asymmetric distribution of these variables, patient groups were compared using the Kruskall-Wallis test followed by Dunn's multiple comparison test.

## Results

A total of 57 patients were included in this study.

### Demographic data

The median (interquartile range) age of the bitten patients was 27 (16-40) years. Males accounted for 61% of the patients and females for 39% (Table I). The most common age groups were 11-20 and 21-30 years (Table I).

**Table I:** Distribution of patients by age group and gender

Demographic data	Sample size (N=57)	%
Age group (year)		
1 - 10	7	12
11 - 20	11	19
21 - 30	17	30
31 - 40	9	16
41 - 50	9	16
51 - 60	4	7
Gender		
male	35	61
female	22	39
**Total**	**57**	**100**

### Circumstances of the bite and use of conventional care

Of the 57 patients, 41 (72%) sought traditional care before going to the hospital, while the remaining 16 (28%) went directly to the hospital.

The distribution of patients by time to treatment is shown in Figure 1. The median time from bite to admission was 7 hours (IQ: 2 – 48). The majority of bites (35/57 or 65%) occurred in the field.

**Figure 1:** Distribution of patients according to the time between bite and hospital admission

Bite sites were distributed as follows: 47 (82%) on the foot, 8 (14%) on the hand, 1 (2%) on the arm, and 1 (2%) on the buttock.

### Severity classification

We did not observe any patient with neurological disorders suspicious for elapid envenomation. The different clinical manifestations are shown in Table II. Cases of hemorrhagic syndrome were the most common, reflecting viperine envenomation. The median length of hospital stay by clinical form is shown in Table II. The maximum length of hospital stay was 11 days. Clinical form influenced the length of hospital stay (p = 0.003), which was longer in cases of WBCT abnormality without bleeding or bleeding compared to dry bite (p = 0.01 and p = 0.007, respectively). On the other hand, there was no difference in hospital length of stay for WBCT abnormality without bleeding versus bleeding (p > 0.99).

**Tableau II:** Classification and duration of hospitalization according to the severity of bites

Classification	Frequency (N=57)	Median length of stay (IQR) (days)
Dry bite	**7 (12)**	**1 (1 -1)**
Isolated local inflammatory syndrome	**15 (26)**	**3 (2 – 4)**
Abnormal WBCT without bleeding	**11 (20)**	**4 (3 – 8)**
Bleeding	**24 (42)**	**4 (3 – 6)**

Out of a total of 57 patients, we observed two deaths (3%) related to hemorrhage: the first case was a 25-year-old female patient who presented to the emergency department one week after snakebite; death occurred 30 minutes after admission to the hospital. The 2^nd^ case, which occurred 2 hours after admission to the hospital, involved a 17-year-old female patient, 20 weeks pregnant, who was admitted 2 hours after being bitten by *Bitis arietans* (a Viperidae larger than *E. ocellatus)* despite the administration of antivenom.

### Cost of treatment

A total of 108 vials of antivenom were used to treat 45 patients (Table III). Twelve patients (seven dry bites and five inflammatory syndromes with local edema) received only symptomatic treatment because their clinical condition did not require antivenom. Cases of hemorrhagic syndrome or abnormal WBCT sometimes required four to six vials of antivenom.

**Tableau III:** Number of vials of antivenom and median cost according to the severity of the envenomation

Classification	No. of patients bitten	No. of vials used	Median number of vials (IQR)	Median cost (IQR) in euros
Dry bite	7	0	0 (0 - 0)	31 (26 - 47)
Isolated local inflammatory syndrome	15	25	2 (2 - 2)	163 (149 - 170)
Abnormal WBCT without bleeding	II	24	2 (2 - 2)	177 (161 - 327)
Bleeding	24	59	2 (2 - 2)	179 (154 - 286)
**Total**	**57**	**108**	**2 (2 - 2)**	

The median costs according to severity are shown in Table III. We observed a significant difference in the cost of treatment according to clinical form (p = 0.0001): compared to a dry bite, the cost of symptomatic forms was higher for isolated local inflammatory syndrome (p > 0.04), WBCT abnormality without bleeding (p = 0.0005), or bleeding (p = 0.0002). There was no significant difference between the median costs of the three symptomatic forms of envenomation (isolated local inflammatory syndrome, WBCT abnormality without hemorrhage, and hemorrhage). Antivenom was the main expense for patient management (Table IV). For each of them, antivenom represented a higher cost than complementary treatments (p = 0.0001), hospitalization (p > 0.0001) and medical-biological tests (p > 0.0001). On a per-patient basis, antivenom accounted for approximately 75% of total treatment costs.

**Tableau IV:** Cost of treatment, total and median per patient

Classification	Total cost (IQ) in euros	Median cost (IQR) per patient in euros
Dry bite	7 115	128 (128 – 128)
Additional treatment	1 264	15 (10 – 30)
Hospitalization	796	12 (8 – 20)
medical-biological tests	817	11 (11 – 15)
**Total**	**9 992**	**168 (154 – 242)**

## Discussion

This prospective study presents a series of patients who strictly adhered to the therapeutic protocol, thus providing a solid basis for estimating the costs of treating snakebite envenomations at the Saint Jean de Dieu Hospital in Tanguiéta.

Direct costs are those of care, consultations, medication, hospitalization, transportation, etc. Indirect costs may include loss of productivity due to hospitalization time, for patients and their families, and for their country due to the high number of envenomations. Few studies conducted in rural Africa have examined the economic burden of envenomations [9, 10], and none, to our knowledge, have examined the direct costs of envenomation management at the patient level. Our study was conducted in an area endemic for *E. ocellatus* bites, during the period of the year when the highest number of cases is recorded [1]. It was conducted exclusively at the hospital level and covered only the first few days of envenomation, the effects of which can last for several weeks.

The high cost, which varies depending on the antivenom available, is rarely affordable for rural populations in sub-Saharan Africa [6, 11, 13, 14, 15, 16]. In our study, antivenom immunotherapy cost an average of€128, representing 76% of the treatment. In Benin, where the minimum monthly wage is €78 [18] and the average monthly per capita income is €95 in 2018 [20], the cost of hospital treatment was almost twice the average monthly per capita income. Nevertheless, all patients whose condition required antivenom received it. The Tanguiéta Hospital is a faith-based hospital that offers payment options to patients and their families. This arrangement ensures that the treatment follows the therapeutic protocol while limiting financial consequences.

In sub-Saharan Africa, patients and their families must pay the entire cost before receiving treatment, which often increases the waiting period. The cost of treating envenomations is often difficult for patients and their families to afford [12]. In a study in Nigeria, one family reported that it would take an entire year to repay the debt incurred, while other families were forced to sell livestock to finance treatment [8]. This economic impact exacerbates poverty, which in turn is a factor in increased mortality, leading to a vicious cycle [19]. During or after hospitalization, when patients are about to return home, they do their best to pay the full cost. In addition to the cultural and mystical reasons that guide the treatment-seeking behavior of a snakebite victim, financial and social consequences have a strong influence on delaying or even refusing to consult a health center.

In a retrospective study conducted in a referral hospital in Kenya, Okumu *et al.* found that the median cost of treatment was $26 (€24) [14]. However, this relatively low cost was associated with a frequent shortage of antivenom in this hospital. Out of 127 bitten patients, only 42% had received antivenom usually at a dose of one vial. In Tanguiéta, no antivenom shortages were reported during the study period.

The study by Okumu *et al.* also noted that longer hospital stays were associated with higher treatment costs [14]. This may also be an incentive to leave hospital against medical advice, when the patient's condition still requires care. Hospitalization time, and therefore cost, may be increased in cases of severe envenomation. However, in our study, there was no significant difference between the costs corresponding to the different clinical forms. On the other hand, it should be noted that the algorithm recommended by the African Society of Toxinology provides for only one vial of antivenom in the case of isolated local syndrome, whereas the patients received two vials, which unnecessarily increased the cost of management [3].

The total burden of envenomations in West Africa has been estimated at 320,000 DALYs (disability-adjusted life years)/year (95% CI: 248,000-403,000) [9]. With the high cost-effectiveness of antivenom use, it is vital for policymakers and governments in sub-Saharan African countries to put in place a mechanism for price reduction or financial compensation [7, 11, 15, 17]. According to O’Brian *et al.* in Mozambique, the median individual cost of snakebite due to loss of income is $17 - not including treatment, notably antivenom, which was not quantified in this study - five times higher than the cost of treating a case of uncomplicated malaria [13]. However, envenomations receive little attention from decision-makers. In Benin, as in other sub-Saharan African countries, there are mechanisms for free care for certain eventualities (malaria, caesarean section, etc.), which could be extended to envenomations. While waiting for the free care advocated by several authors [7, 16], we can propose a fair distribution of costs based on two driving forces. Firstly, the cost of antivenoms could be shared between the state, which, in addition to a subsidy, would centralize orders to benefit economies of scale; local authorities, which would estimate needs and manage regional stocks; and private companies, which would contribute to the payment of the bill, leaving patients with a manageable out-of-pocket expense. On the other hand, hospitals could charge a flat fee to share the burden among patients regardless of the severity of their poisoning.

## Limitations

Our study was limited to direct medical costs. It would have been difficult to evaluate direct non-medical costs (transport, food, etc.). For example, patients were brought to the hospital by their own means of transportation, usually a motorcycle, or borrowed –perhaps rented– from the community. Our study measured only the costs incurred by patients. It would be interesting to show the actual costs incurred by the hospital (cost of antivenom serum, other drugs, food, payment of staff, etc.).

In addition, the indirect costs (e.g. absence from work) borne by the patient's caregivers could not be assessed due to the difficulty of the task; many of them leave their jobs to be at the side of the patient (community solidarity). The absence of a community member leads to a reorganization of the sharing of activities within the family or village [5]. Mutual aid is an inexpensive form of substitution, the aim being to avoid the need for a paid substitute. It is still very active among community members and helps to reduce overhead costs.

This study was conducted as part of a Phase 4 clinical trial to evaluate the efficacy and safety of tranexamic acid in combination with antivenom in the treatment of hemorrhage associated with snakebite envenomation (ACTRASES study). Of the 45 patients treated with antivenom, 14 were enrolled in this trial, with most of their costs covered. This may have artificially inflated the cost of antivenoms in our study, which may not have been purchased if the patient had to pay for them. Finally, this study did not allow us to describe the traditional treatments that patients were receiving or to assess their potential benefits and risks. In addition to the delay in treatment due to the consultation of a traditional healer, the gestures performed by the latter, such as the application of tourniquets, skin incisions or the administration of emetic plants to slow down the diffusion of the poison or to promote its elimination, could be harmful and lead to longer hospital stays and higher treatment costs.

## Conclusion

Our study confirmed that snakebite envenomation is costly, sometimes exceeding the average monthly income of many households. In the case of hemorrhage, the cost is further increased by the need to renew antivenom vials. These bites occur in rural areas, among a poor population engaged in agropastoral activities with rudimentary means. They affect young people, who are the most productive segment of society. Snakebite envenomation, linked to agricultural activities, affect them, leading to a decrease in productivity and an additional risk of impoverishment. The cost of treatment is borne entirely by the patients and their families, reinforcing the vicious circle of poverty.

It is therefore desirable to establish a mechanism to finance the treatment of envenomations. As an alternative to providing antivenoms free of charge to patients, the costs could be shared among all stakeholders. Reducing the cost of antivenoms will allow better access to hospital care and early treatment of patients, thereby reducing the economic burden of envenomations.

## Acknowledgements

We would like to thank all the staff of the Saint Jean de Dieu Hospital in Tanguiéta for their hospitality and assistance during this study. We are also grateful to the patients and their families for their participation and understanding during our interviews. We would also like to thank the *Institut de Recherche Clinique du Bénin* for allowing us to conduct this study concurrently with the ACTRASES clinical trial.

## Funding

Funding for this study was shared between the *Institut de Recherche pour le Développement* and the *Institut de Recherche Clinique du Bénin* (logistics), Senghor University, Alexandria, Egypt (accommodation cost of N. Tourita) and Inosan Biopharma (supply of antivenom for the ACTRASES study).

## Authors’ contributions

NT: study design, protocol writing, data collection, data analysis, manuscript writing

NS: data collection, correction of manuscript

SAO: data collection, verification and validation of results, correction of manuscript

EG: data collection, correction of manuscript

AM: study sponsor, revision and validation of protocol, correction of manuscript

JPC: acquisition of funding, review and validation of protocol, validation of data, validation of analysis, review and correction of manuscript

SL: study design, drafting and validation of protocol, study coordination, verification and validation of data, verification of analysis, revision and correction of manuscript

## Conflict of interest

The authors declare no conflict of interests.
